# Trust in Government, Perceived Integrity and Food Safety Protective Behavior: The Mediating Role of Risk Perception

**DOI:** 10.3389/ijph.2023.1605432

**Published:** 2023-03-08

**Authors:** Jie Liu, Ziqiang Han, Yihong Liu, Sombo William

**Affiliations:** ^1^ School of Political Science and Public Administration, Shandong University, Jinan, China; ^2^ School of Public Administration and Policy, Renmin University of China, Beijing, China

**Keywords:** risk perception, food safety, protective behaviors, perceived integrity, trust in government

## Abstract

**Objective:** This study examined the correlations between trust in government and the public’s protective behaviors regarding food safety, focusing on the mediating role of risk perception.

**Methods:** The 2013 (1,432 samples) and 2019 (1,276 samples) Taiwan Social Change Survey data were analyzed using ordinary least squares regression models. The bootstrap method was used to examine the mediating effect of risk perception.

**Results:** Perceived integrity of government regarding food safety issues influences all four types of food protective behaviors directly and indirectly *via* risk perceptions. The four protective behaviors were “not eating that food,” “preparing food kit,” “preferring organic food,” and “overall behaviors.” Trust in government directly influences part of the protective food behaviors, while no mediating effects of risk perception were found.

**Conclusion:** The results of this study will deepen our understanding of food consumption behavior, identify key factors that influence public food protective behaviors, and inform food safety management to implement strategies necessary to improve food consumption.

## Introduction

Food safety is one of the most complex and concerned social issues in China (1). In 2008, a nationwide incident of contaminated infant formula resulted in over 290,000 children suffering from urinary tract stones, and this event triggered public and media’s concern of food safety, and these impacts have lasted decades (2). Within Taiwan region, a series of food scandals, either large or small, also emerged in recent years (3). The lay public, which has a zero-tolerance attitude toward food safety, may be alarmed by these food safety-related events. Nevertheless, with the exception of the melamine incident, the health effects of most of these events have largely been under-assessed (4). Food safety regulation agencies and media are the primary sources of food safety information for the public, and they heavily influence the public’s risk perception and protective behaviors (5, 6). Such connection, however, has not received sufficient attention in the food safety field. To address this knowledge gap, we employed two cross-sectional representative surveys from Taiwan to examine the association between trust in government, the perceived integrity of government regarding food safety issues, and food protection behaviors, focusing on the mediating role of public perceptions of food safety.

Understanding the public’s food consumption and self-protective behaviors in the face of food risk is particularly important for food safety management and risk regulation in the food industry. Therefore, it is essential to investigate the protective behaviors concerning food consumption from the behavioral science perspective. Generally, models such as the health belief model, the theory of planned behavior, the social-cognitive model (7–10), and the protective action decision model (11) are developed by scholars to facilitate the understanding of human behaviors about risks. Food choices are often influenced more by psychological interpretations of product characteristics than by the product’s physical characteristics (12). Risk perception is one such interpretation. Risk perception thus has implications for the purchase and production behavior of consumers and producers, as well as the overall effectiveness and efficiency of the food supply chain. When there are deficiencies in risk communication, or when consumers lose confidence or trust in the food supply chain and its various agents, especially the government, there will be a considerable gap between objective technical risk and subjective psychological risk (13).

The attributes of the information that individuals receive and their comprehension of the information can influence their risk perceptions and their self-protective actions (14). The attributes of the information can include sources, channels, formats, and the receivers’ trust in this information (11). The comprehension process varies according to the individual’s competence and the characteristics of the information (15). Risk communication sources from authorities, experts, media, and individual social networks can be perceived, interpreted, amplified, or attenuated differently (14, 16–18). The degree of trust in information sources influences risk perception and behavioral response, especially when individuals lack professional knowledge regarding a specific risk or are in a situation of uncertainty (19–21). Indeed, trust in information sources can reduce the degree of the perceived risk if the information’s purpose is to reduce the public’s concern about risk (22–24).

In the food safety context, the perception of food safety risks as consumers’ beliefs determine the consumers’ intentions and behaviors of purchasing those food products (19, 25–27). Consumers usually adjust their purchasing decisions to alleviate the risks by reducing, shifting, or postponing the purchase of the offending products if unavoidable (28) or seeking advice from trusted sources (13). Trust can be characterized as relying on those responsible for managing public health and safety (24). Considering the diverse sources of food safety and food quality information, trust in different information sources can vary, and affect the consumer to evaluate the information, especially when they lack appropriate knowledge (29). A previous study showed that information from government and state authorities could be a reliable source if the government were perceived as a neutral agency in most cases (30–32). Government and authorities play an essential role that could influence the consumers’ perception of food safety and quality and eventually affect their consumption behaviors (33). The above leads to the following hypothesis:

H1 Trust in government directly predicts the degree of taking food protective behaviors. With a higher trust degree of government, people would have a larger likelihood to take protective behaviors to protect themselves.

Similarly, many aspects of life have been taken out of the control of the individual, including access to the food production system, which requires reliance on and trust in external institutions (34). Decisions about food risks are difficult for consumers if the information is not readily available and there are no alternatives to foods that are considered potentially risky (35). Factors influencing food choices often include government policies supporting specific agricultural or industrial practices. Thus, consumers select their purchases based on their reliance on and confidence in relevant government institutions (10, 36–40). Information uncertainty can lead to public mistrust and confusion (41). A Swedish case showed that the public became unnecessarily worried and confused as regulators magnified potential food risks that should have been mitigated (42). Citizens tend to distrust the government if they perceive that authorities often or sometimes keep vital public interest or information secret (43) and instead act in their personal best interests, also known as individualization (44). So, it is critical to note that the high perceived integrity of government regarding food safety issues leads to a sense of predictability (45) and a perception of low risk (46). Therefore, we hypothesize:

H2 The perceived integrity of government regarding food safety issues directly influences the public’s adoption of food protective behaviors. With less fear that the government hides food safety information, people would have less willingness to take protective behaviors.

Risk perception also significantly influences people’s food consumption behaviors (47). It may not be surprising that in the food safety domain, risk perception is considered to be a determinant of attitudes and behaviors (48–50). This rationale is also in line with influential theoretical models such as the theory of planned behavior (7, 8). Individuals’ judgments and behavioral adoptions are also influenced by their trust in those responsible for risk assessment and regulation, especially when they cannot judge the risk k on their own, as in the case of food safety issues (24, 51–53). Thus, perceptions of the trustworthiness of those responsible for approving and regulating food may impact people’s perceptions of risks and their acceptance. The above makes it reasonable to assume that the public’s trust and perceived integrity in authority influences their risk perceptions and, thus, their food consumption behaviors. Therefore, after combining H1 and H2, we hypothesize that:

H3 Risk perception of food safety mediates the relationship between trust and “not eating” (H3a), “preparing food kit” (H3b), “preferring organic food” (H3c), and “overall behaviors” (H3d).

H4 Risk perception of food safety mediates the relationship between the perceived integrity of government regarding food safety issues and “not eating” (H4a), “preparing food kit” (H4b), “preferring organic food” (H4c), “overall behaviors” (H4d).

**FIGURE 1:**
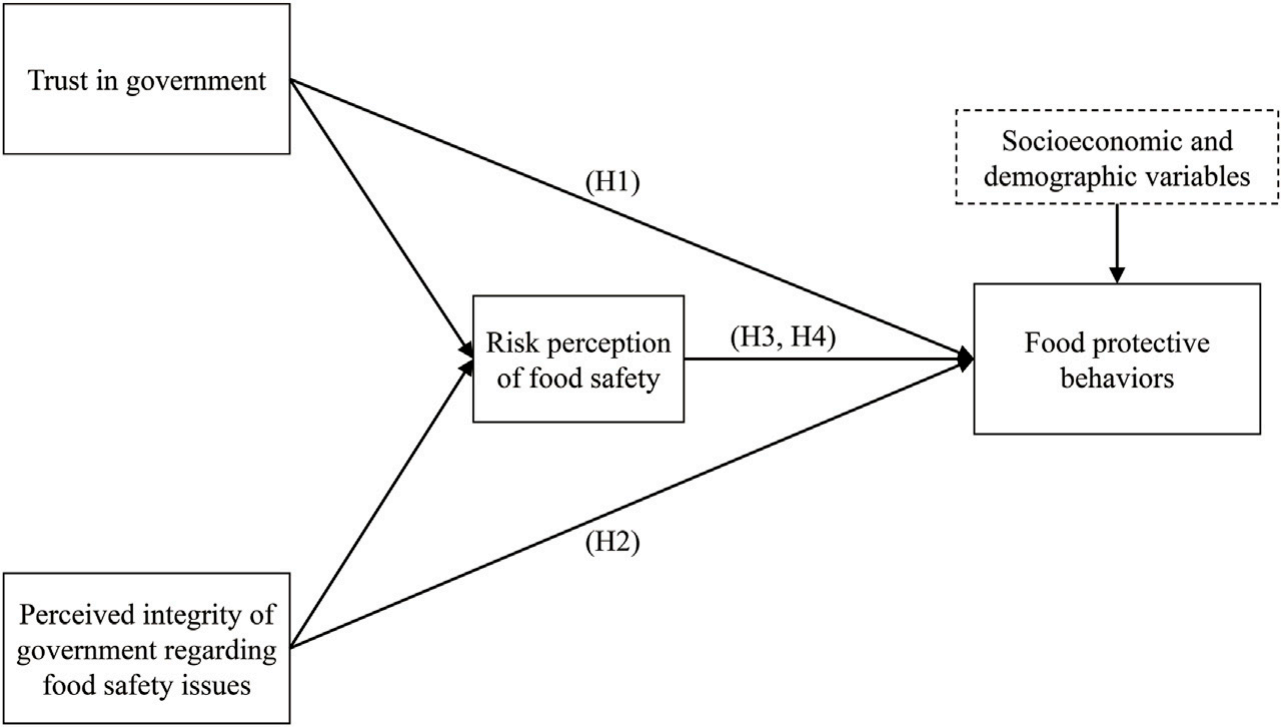
The hypothesized model framework (Taiwan Social Change Survey, Taiwan region, 2013 and 2019).

Additionally, individuals’ socioeconomic and demographic factors were incorporated into the hypothesized model as control variables. Figure 1 illustrates the hypothesized model framework.

## Methods

### Data and Sampling

This paper analyzed the 2013 and 2019 Taiwan Social Change Survey (TSCS) risk society module data, respectively. The TSCS survey is a representative repeated cross-sectional survey conducted annually by the Institute of Social Science of Academia Sinica in collaboration with the Humanities and Social Sciences Research Center in Taiwan. The data is collected by face-to face interviews and publicly available from the Academia Sinica’s Survey Research Data Archive website and open to registered researchers.

Risk society modules were included in the 2013 and 2019 surveys. According to the reports of both surveys, the stratified three-stage (town-community-individual) probability and proportional-to-size sampling method were employed for both, and the 2013 data included 2,005 individuals, while the 2019 data covered 1,933 samples. 1,432 observations from the 2013 data and 1,276 samples from the 2019 data were included in the models because the ones with missing values were dropped directly in the analysis.

### Measurement

#### Dependent Variables

The intentions for adopting protective behaviors are used as the proxy of actual behaviors because it is very difficult to measure the actual behaviors in reality (54, 55). Four food safety protective behaviors were used in this survey: “If media (i.e., media in the food and health sector) reported that there would be food safety issues, will you try to avoid eating it?”; “Will you bring your food kit when you eat out due to food safety concerns?”; “Will you try your best to eat organic food?” In a previous study of food safety in Taiwan, organic food was defined as food that is not genetically modified and produced naturally, especially without using synthetic chemicals such as pesticides and chemical fertilizers (56). Each question was measured on a 4-point Likert scale (1 = definitely not, 4 = definitely will). The sum of the three variables was then included in the analysis as the fourth one, “overall behaviors” (Cronbach’s alpha = 0.71 in 2013; Cronbach’s alpha = 0.62 in 2019).

#### Independent Variables

The trust in the government concept was measured in two dimensions. The first one was directly captured by the question, “In general, do you trust the government?” The “central/provincial government,” “county government,” and “town government” were listed as different levels of government. The trust in each type of government was represented on a 5-point Likert scale (1 = complete distrust, 5 = complete trust). The average of the three variables was used as the degree of trust in government (Cronbach’s alpha = 0.74 in 2013; Cronbach’s alpha = 0.78 in 2019).

The perceived integrity of government in the food safety area was used as the second dimension of the trust in government, and it was measured by the question, “Are you concerned that the government hides food safety information?” A 5-point Likert scale measured the answers (1 = very concerned, 5 = very little concerned), indicating an increased degree of integrity of government regarding food safety issues.

#### Mediating Variable

The risk perception for food safety was calculated from the responses collected from three questions: “How do you concern about possible pollution from heavy metal or plasticizers?,” “How do you concern about the pesticide residue in fruit, vegetables, and grocery?” and “How do you concern humans using biotechnology to alter the genes of plants and animals?” A 5-scale Likert scale measured the responses to each of the three questions (1 = very little concerned, 5 = very concerned), indicating the increased degree of concern. The sum of the responses to all three questions was used as the risk perception score in this analysis (Cronbach’s alpha = 0.71 in 2013; Cronbach’s alpha = 0.83 in 2019).

#### Control Variables

In reference to prior studies on risk perception and food protective behaviors (57–63), the ages of the respondents, gender, education attainments, religious status, current job status, marital status, whether having children at home, self-perceived social status, and the geographical locations of the respondents were included as control variables. See Table 1 for more information on the categories.

**TABLE 1:** Characteristics of sample (Taiwan Social Change Survey, Taiwan region, 2013 and 2019).

Variable	2013			2019		
	Mean (SD)	Min	Max	Mean (SD)	Min	Max
Protective adaptations	8.45 (1.92)	3	12	8.58 (1.83)	3	12
Trust in government general	3.30 (0.79)	1	5	3.26 (0.81)	1	5
Perceived integrity of government	1.86 (0.98)	1	5	1.93 (1.02)	1	5
Risk perception	11.46 (2.41)	3	15	11.50 (2.68)	3	15
Perceived social status	4.67 (1.73)	1	10	5.08 (1.69)	1	10
Age	49.93 (15.91)			46.65 (16.15)		
	Freq.	Percent	Cum.	Freq.	Percent	Cum.
Not eat						
Definitely not	83	5.80	5.80	48	3.76	3.76
Probability not	215	15.01	20.81	178	13.95	17.71
Probability will	540	37.71	58.52	505	39.58	57.29
Definitely will	594	41.48	100	545	42.71	100
Prepare food kit						
Definitely not	208	14.53	14.53	201	15.75	15.75
Probability not	415	28.98	43.51	298	23.35	39.11
Probability will	474	33.10	76.61	440	34.48	73.59
Definitely will	335	23.39	100	337	26.41	100
Prefer organic food						
Definitely not	113	7.89	7.89	110	8.62	8.62
Probability not	484	33.80	41.69	400	31.35	39.97
Probability will	635	44.34	86.03	594	46.55	86.52
Definitely will	200	13.97	100	172	13.48	100
Gender						
Female	658	45.95	45.95	582	45.61	45.61
Male	774	54.05	100	694	54.39	100
Education						
Primary	187	13.06	13.06	143	11.21	11.21
Middle	142	9.92	22.97	120	9.40	20.61
High	122	8.52	31.49	129	10.11	30.72
College+	981	68.50	100	884	69.28	100
Having children at home						
No	844	58.94	58.94	832	65.20	65.20
Yes	588	41.06	100	444	34.80	100
Religion						
No	291	20.32	20.32	341	26.72	26.72
Religion	1,141	79.68	100	935	73.28	100
Job status						
Fulltime	829	57.89	57.89	808	63.32	63.32
Part-time	173	12.08	69.97	71	5.56	68.89
Jobless	4.3	3.00	72.97	39	3.06	71.94
Students	86	6.01	78.98	57	4.47	76.41
Housework	301	21.02	100	301	23.59	100
Marriage						
Single	389	27.16	27.16	348	27.27	27.27
Married	922	64.39	91.55	780	61.13	88.40
Divorced	70	4.89	96.44	86	6.74	95.14
Widowed	51	3.56	100	62	4.86	100
Location						
Mega cities	300	20.12	20.12	311	24.37	24.37
Middle size cities	405	27.16	47.28	372	29.15	53.53
New towns	456	30.58	77.87	295	23.12	76.65
Traditional towns	132	8.85	86.72	142	11.13	87.77
General towns	128	8.58	95.31	80	6.27	94.04
Aged/remote towns	70	4.69	100	76	5.96	100
Number of observations	1,432			1,276		

### Analytical Strategies

The descriptive analysis was first demonstrated. For the indicators constructed by more than one variable, Cronbach’s alpha test was conducted to explore the internal consistency of treating the several variables as one. The Ordinary Least Squares (OLS) regression models were employed when the protective behaviors were used separately. Simple mediation models regarding trust in government and perceived integrity of government regarding food safety which affects food safety protective behaviors *via* risk perceptions, were assessed (64). All the analyses were implemented by Stata/SE 15.1 version.

## Results

### The Characteristics of the Sample

1,432 respondents were included in the 2013 TSCS, 54.1% were male, 79.7% had religious beliefs, and 41.1% had a child(ren) at home. In the 2019 data, 1,276 respondents were included, 54.4% were male, 73.3% had religious beliefs, and 34.8% had a child(ren) at home, respectively. Respondents generally had lower perceived integrity of government regarding food safety issues, both in 2013 (M = 1.9, SD = 1.0) and 2019 (M = 1.9, SD = 1.0). Nevertheless, they had a relatively higher level of trust in the government, reaching 3.3 in both the 2013 and 2019 surveys. Risk perception maintained similar levels in 2013 and 2019. Regarding the three food protective behaviors, the percentage of “definitely not,” “probability not,” “probability will,” and “definitely will” eating the food if a news report about the food safety issue were 5.8%, 15.0%, 37.7%, 41.5% in the 2013 survey, while these distributions in the 2019 survey were 3.8%, 14.0%, 39.6%, 42.7% in the 2019 survey. Similarly, the “preparing food kit” choices from “definitely not” to “definitely will” were 14.5%, 29.0%, 33.1%, and 23.4% in the 2013 survey, while they were 15.8%, 23.4%, 34.5%, 26.4% in the 2019 survey. The shares of “preferring organic food” in the 2013 survey were 7.9% (definitely not), 33.8% (probability not), 44.3% (probability will), and 14.0% (definitely will), and these percentages in the 2019 survey were 8.6%, 31.4%, 46.6%, 13.5%, respectively. Table 1 reported all the descriptive analyses of the respondents.

### Direct effects of trust and perceived integrity on food protective behaviors

H1 examined whether trust in the government directly influenced food protective behaviors. The results show that trust has a significant direct association with “not eating that food” (*β* = 0.08, *p* < 0.01 in 2013; *β* = 0.09, *p* < 0.01 in 2019), “preparing food kit” (*β* = 0.08, *p* < 0.05 in 2013; *β* = 0.11, *p* < 0.01 in 2019), “preferring organic food” (*β* = 0.11, *p* < 0.001 in 2013; *β* = 0.06, *p* < 0.05 in 2019), and “overall behaviors” (*β* = 0.27, *p* < 0.001 in 2013; *β* = 0.26, *p* < 0.001 in 2019) (Tables 2, 3). Thus, H1 was supported.

**TABLE 2:** Direct effects of trust in government and perceived integrity of government regarding food safety issues on adopting protective behaviors (Taiwan Social Change Survey, Taiwan region, 2013).

	Risk perception	Not eat	Prepare	Organic	Overall behaviors
Risk perception		0.10*** (0.01)	0.09*** (0.01)	0.07*** (0.01)	0.26*** (0.02)
Trust in government general	0.06 (0.07)	0.08** (0.03)	0.08* (0.03)	0.11*** (0.03)	0.27*** (0.06)
Perceived integrity of government	−0.84*** (0.06)	−0.06* (0.02)	−0.09** (0.03)	−0.11*** (0.02)	−0.25*** (0.05)
Job (fulltime as reference)					
Part-time	0.22 (0.19)	0.005 (0.07)	0.03 (0.08)	−0.04 (0.07)	−0.01 (0.14)
Jobless	0.14 (0.35)	0.10 (0.13)	0.16 (0.14)	0.22^+^ (0.12)	0.49^+^ (0.27)
Students	−0.04 (0.27)	−0.19^+^ (0.10)	−0.08 (0.11)	−0.03 (0.10)	−0.30 (0.21)
Housework	0.12 (0.18)	−0.03 (0.07)	−0.09 (0.08)	−0.02 (0.07)	−0.14 (0.14)
Education (primary as reference)					
Middle	0.10 (0.26)	0.06 (0.10)	−0.10 (0.11)	0.03 (0.09)	−0.01 (0.20)
High	0.19 (0.28)	0.01 (0.10)	0.10 (0.11)	0.17^+^ (0.10)	0.28 (0.21)
College+	0.20 (0.22)	0.10 (0.08)	0.43*** (0.09)	0.18* (0.08)	0.71*** (0.17)
Marriage (single as reference)					
Married	0.57** (0.19)	0.20** (0.07)	0.15^+^ (0.08)	0.10 (0.07)	0.45** (0.15)
Divorced	0.06 (0.31)	0.16 (0.12)	−0.005 (0.13)	−0.06 (0.11)	0.10 (0.24)
Widowed	−0.20 (0.39)	0.43** (0.15)	0.11 (0.16)	0.05 (0.14)	0.59* (0.30)
Location (megacities as reference)					
Middle size cities	−0.22 (0.17)	−0.03 (0.06)	−0.17* (0.07)	0.12* (0.06)	−0.08 (0.13)
New towns	0.08 (0.17)	−0.16* (0.06)	−0.12^+^ (0.07)	0.08 (0.06)	−0.20 (0.13)
Traditional towns	0.03 (0.24)	−0.05 (0.09)	0.07 (0.10)	0.26** (0.09)	0.28 (0.19)
General towns	−0.32 (0.24)	−0.08 (0.09)	0.06 (0.10)	0.22** (0.08)	0.20 (0.18)
Aged/remote towns	0.36 (0.30)	−0.02 (0.11)	−0.10 (0.13)	0.08^+^ (0.11)	0.07 (0.23)
Children	−0.31* (0.13)	−0.02 (0.05)	−0.04 (0.05)	0.03 (0.05)	−0.03 (0.10)
Perceived status	0.02 (0.04)	0.01 (0.01)	0.05** (0.01)	0.04** (0.01)	0.10*** (0.03)
Gender (female as reference)	−0.68*** (0.12)	−0.03 (0.05)	−0.37*** (0.05)	0.03 (0.04)	−0.37*** (0.09)
Age	0.01^+^ (0.01)	0.0002 (0.002)	0.005^+^ (0.003)	0.004^+^ (0.002)	0.009^+^ (0.005)
Religion (None as reference)	0.27^+^ (0.15)	0.05 (0.06)	0.03 (0.06)	−0.02 (0.05)	0.06 (0.12)

Beta values are presented. Standard errors in parentheses. ^+^ *p* < 0.10, * *p* < 0.05, ** *p* < 0.01, *** *p* < 0.001.

**TABLE 3:** Direct effects of trust in government and perceived integrity of government regarding food safety issues on adopting protective behaviors (Taiwan Social Change Survey, Taiwan region, 2019).

	Risk perception	Not eat	Prepare	Organic	Overall behaviors
Risk perception		0.05*** (0.01)	0.08*** (0.01)	0.07*** (0.01)	0.20*** (0.02)
Trust in government general	0.08 (0.08)	0.09** (0.03)	0.11** (0.03)	0.06* (0.03)	0.26*** (0.06)
Perceived integrity of government	−1.00*** (0.07)	−0.08*** (0.03)	−0.11*** (0.03)	−0.07*** (0.02)	−0.26*** (0.05)
Job (fulltime as reference)					
Part-time	0.05 (0.29)	0.06 (0.10)	−0.05 (0.12)	0.02 (0.10)	0.03 (0.20)
Jobless	−0.38 (0.39)	0.20 (0.13)	−0.05 (0.15)	−0.002 (0.13)	0.15 (0.27)
Students	−0.40 (0.35)	−0.05 (0.12)	0.02 (0.14)	0.02 (0.12)	−0.004 (0.24)
Housework	0.02 (0.20)	0.12^+^ (0.07)	0.13 (0.08)	−0.03 (0.07)	0.22(0.14)
Education (primary as reference)					
Middle	0.23 (0.31)	0.04 (0.10)	0.33** (0.12)	0.07 (0.10)	0.44* (0.21)
High	0.95** (0.32)	−0.05 (0.11)	0.47*** (0.12)	0.11 (0.10)	0.53* (0.22)
College+	0.63* (0.27)	0.08 (0.09)	0.65*** (0.10)	0.16^+^ (0.09)	0.89*** (0.18)
Marriage (single as reference)					
Married	0.88*** (0.21)	0.09 (0.07)	0.08 (0.08)	0.004 (0.07)	0.18 (0.14)
Divorced	0.23 (0.32)	−0.02 (0.11)	−0.21^+^ (0.12)	−0.09 (0.10)	−0.32 (0.22)
Widowed	0.57 (0.39)	0.05 (0.13)	0.27^+^ (0.15)	0.02 (0.13)	0.35 (0.26)
Location (megacities as reference)					
Middle size cities	0.16 (0.18)	0.02 (0.06)	−0.19** (0.07)	0.04 (0.06)	−0.14 (0.12)
New towns	0.07 (0.19)	0.01 (0.06)	−0.24** (0.07)	−0.04 (0.06)	−0.27* (0.13)
Traditional towns	0.29 (0.24)	0.16^+^ (0.08)	−0.31** (0.10)	0.11 (0.08)	−0.04 (0.16)
General towns	−0.65* (0.30)	0.35*** (0.10)	−0.20^+^ (0.10)	0.05 (0.10)	0.20 (0.21)
Aged/remote towns	0.11 (0.31)	0.17 (0.10)	−0.42*** (0.13)	0.07 (0.10)	−0.19 (0.21)
Children	0.28^+^ (0.15)	−0.03 (0.05)	−0.003 (0.06)	0.01 (0.05)	−0.02 (0.10)
Perceived status	0.03 (0.04)	0.001 (0.01)	−0.03^+^ (0.02)	−0.06*** (0.01)	−0.09** (0.03)
Gender (female as reference)	−0.74*** (0.14)	0.07 (0.05)	−0.33*** (0.05)	−0.01 (0.05)	−0.27** (0.09)
Age	0.01 (0.01)	0.004 (0.002)	0.001 (0.003)	0.01** (0.002)	0.01* (0.005)
Religion (None as reference)	0.33* (0.15)	0.09^+^ (0.05)	0.05 (0.06)	0.02 (0.05)	0.15 (0.11)

Beta values are presented. Standard errors in parentheses. ^+^ *p* < 0.10, * *p* < 0.05, ** *p* < 0.01, *** *p* < 0.001.

H2 hypothesized that the perceived integrity of government regarding food safety issues would be directly related to food protective behaviors. As we hypothesized, perceived integrity was significantly and negatively associated with all four protective behaviors in both 2013 and 2019, supporting H2.

### The Mediating Effects of Risk Perception

The bootstrapping method (65) was used to examine the mediating effects of risk perception (H3 and H4). The indirect effects of trust and perceived integrity on the four dependent variables with risk perception as a mediator were tested (Table 4). Before that, we tested whether trust in government and perceived integrity were associated with risk perception. The results showed that perceived integrity was significantly related to risk perception, both in 2013 and 2019, while no correlation was found between trust in government and risk perception (Tables 2, 3). All control variables were included in all analyses.

**TABLE 4:** Indirect effects of trust in government on protective behaviors *via* risk perception (Taiwan Social Change Survey, Taiwan region, 2013 and 2019).

	2013			2019		
	Estimation (SE)	95% CI	% of mediation	Estimation (SE)	95% CI	% of mediation
Trust in government →						
Not eat	0.01 (0.01)	[−0.01, 0.02]	0.11	0.004 (0.004)	[−0.005, 0.01]	0.04
Prepare food kit	0.005 (0.01)	[−0.01, 0.02]	0.06	0.01 (0.01)	[−0.01, 0.02]	0.08
Prefer organic food	0.004 (0.01)	[−0.01, 0.01]	0.04	0.005 (0.01)	[−0.01, 0.02]	0.08
Overall behaviors	0.01 (0.02)	[−0.02, 0.05]	0.04	0.01 (0.02)	[−0.02, 0.05]	0.04
Perceived integrity →						
Not eat	−0.09 (0.01)^a^	[−0.11, −0.06]	0.60	−0.05 (0.01)^a^	[−0.07, −0.03]	0.38
Prepare food kit	−0.07 (0.01)^a^	[−0.09, −0.05]	0.50	−0.08 (0.01)^a^	[−0.10, −0.06]	0.42
Prefer organic food	−0.06 (0.01)^a^	[−0.07, −0.04]	0.40	−0.07 (0.01)^a^	[−0.09, −0.05]	0.50
Overall behaviors	−0.21 (0.02)^a^	[−0.26, −0.17]	0.46	−0.20 (0.02)^a^	[−0.24, −0.15]	0.43

Bias-corrected 95% confidence intervals based on 5,000 bootstrap samples.

^a^ CI did not encompass zero, indicating mediation is assumed.

Tests of indirect effects to assess mediation revealed that risk perception did not mediate the correlations between trust in government and any type of protective behaviors, either in 2013 or 2019. Hence, H3a, H3b, H3c, and H3d were not supported (Table 4).

Risk perception mediated the relationship between perceived integrity on “not eating that food” (*β* = −0.09, 95% CI = [−0.11, −0.06] in 2013; *β* = −0.05, 95% CI = [−0.07, −0.03] in 2019), “preparing food kit” (*β* = −0.07, 95% CI = [−0.09, −0.05] in 2013; *β* = −0.08, 95% CI = [−0.10, −0.06] in 2019), “preferring organic food” (*β* = −0.06, 95% CI = [−0.07, −0.04] in 2013; *β* = −0.07, 95% CI = [−0.09, −0.05] in 2019), and “overall behaviors” (*β* = −0.21, 95% CI = [−0.26, −0.17] in 2013; *β* = −0.20, 95% CI = [−0.24, −0.15] in 2019), indicating that perceived integrity influences food protective behaviors both directly and indirectly. Therefore, H4a, H4b, H4c, and H4d were supported.

### The Roles of Control Variables

Age of the public was a significant positive predictor of “preferring organic food” and “overall behaviors” in 2019. Respondents with college education and above were more active in adopting “preparing food kit” and “overall behaviors” both in 2013 and 2019, suggesting that well-educated persons tend to care about food safety risk than their less-educated counterparts. Meanwhile, Men are less inclined to adopt “preparing food kit” and “overall behaviors” than women, both in 2013 and 2019, which women may be more concerned about food safety and attach greater importance to healthy eating (66). Participants with higher self-rated social status in 2013 were more likely to adopt “preferring organic food” and “overall behaviors,” yet the opposite result was observed by 2019. We speculate that this may be related to socioeconomic changes, where the wealthy have easier access to quality material resources than before, which makes them less concerned about food safety. Additionally, those who were married or widowed were more likely to adopt “not eating that food” and “overall behaviors” in 2013, while no similar results were found in 2019.

## Discussion

This study investigated the risk concerns on food safety based on the trust in government and perceived integrity of government regarding food safety issues, about their self-protective behaviors. Furthermore, the roles of individual demographic factors were considered to extend our understanding of how information is processed by consumers. The present study allowed attempting to replicate and extend previous research on the perceived risk of food safety. In this section, some theoretical and policy implications for risk communication and future research, plus the strengths and limitations of this study are presented.

Some of the hypothesized paths were found to be highly significant. Firstly, the perceived integrity of a system, say technology or a financial market, comes about when citizens or investors perceive that the information released by regulators is consistent with what they perceived, which leads to a sense of predictability and being in control (45), and the perception of low risk (46). Our analysis revealed that the perceived integrity of government regarding food safety issues influenced the food protective behaviors directly. Meanwhile, complex factors such as human perception, judgment, and choice have evolved to allow decision-makers to function in a broad range of environments that change with seasons or political regimes and over time. Thus, human actors need to procure sustenance regularly, which may require exploration and risk-taking. At the same time, there is the need to ensure safety and survival, which also require protection and caution. When the public has a sufficiently high level of acceptance of food safety information released by the government, their food consumption behavior is easily predicted. This finding has positive implications for urging health officials to be cautious in releasing food information to reassure the public.

Secondly, H1 was fully supported, argued that trust in government directly influences food protective behaviors, although no mediating role of risk perception between the two was found. The presence or absence of trust in government will play an important role in controlling risks. The findings imply that whether these consumer perceptions are accurate or not, they still can affect future consumption decisions and behaviors, the reputation of government institutions and the food industry. Consumers lack the scientific and infrastructural capacity to evaluate food risk. Hence, it is incumbent on the government and its institutions to provide the relevant guidelines and regulations to foodservice actors and ensure their enforcement to protect consumers (67). Past research showed that the degree of trust is significantly related to the attitude toward the potential threat and behavioral expectations of adopting protective behaviors (11, 68–70). These findings suggest that government, as an important food safety regulator, needs to earn a high level of public trust to promote food consumption behavior.

Additionally, risk perception mediated the effects of the perceived integrity of government regarding food safety issues. The mediating role of individual-level risk perception has been demonstrated in previous studies (13, 50, 71). Our findings further expand the literature in this area by revealing that trust in government influences food consumption behavior by affecting public risk perceptions. The results indicated that risk perception partially mediated the relationship between perceived integrity on food protective behaviors. Despite the mediating role of risk perception, perceived integrity still directly influences food protective behaviors, suggesting that government needs to take on a greater role as a food safety communicator. In most cases, the public will trust the regulator, even if they disagree with the regulatory decision, as long as they perceive the process to be credible, i.e., fair, competent, and efficient (41).

This study acknowledges its limitations. In the first place, there are non-negligible limitations in the cross-sectional survey design of this study that do not allow for causal relationships between the variables we analyzed. Secondly, since we used secondary data collected and designed by others, measures of key variables used in the model such as the risk perception could not be as precise as we would have wanted. Thirdly, loss of sample size due to missing values (29% in 2013; 34% in 2019), which may have an impact on the final results. Last but not least, although the relationship between information credibility and risk perception is certainly longstanding, the circumstances in which this relationship is expressed today, present great challenges and as such, an opportunity for future researches. The mechanisms linking credibility, information processing, and risk perception are likely to be located in motivation, issue involvement, information-holding, and the effect of message cues (72). More evidence of the relationship between food safety, risk perception, and protective behavior should be explored in future studies.
